# Histone Methyltransferase DOT1L Is Involved in Larval Molting and Second Stage Nymphal Feeding in *Ornithodoros moubata*

**DOI:** 10.3390/vaccines8020157

**Published:** 2020-04-01

**Authors:** Julia Gobl, Deepak Kumar Sinha, Radek Sima, Jan Perner, Petr Kopáček, James J Valdés, Ryan O. M. Rego, Alejandro Cabezas-Cruz

**Affiliations:** 1 Faculty of Science, University of South Bohemia, 37005 České Budějovice, Czech Republic; gobl.julia96@gmail.com (J.G.); babadeep22@gmail.com (D.K.S.); sima@paru.cas.cz (R.S.); kopajz@paru.cas.cz (P.K.); valdjj@gmail.com (J.J.V.); 2 Institute of Parasitology, Biology Center, Czech Academy of Sciences, 37005 České Budějovice, Czech Republic; perner@paru.cas.cz; 3 Institute of Biological Sciences, SAGE University, Indore 452020, India; 4 Department of Virology, Veterinary Research Institute, Hudcova 70, 62100 Brno, Czech Republic; 5 UMR BIPAR, INRAE, ANSES, Ecole Nationale Vétérinaire d’Alfort, Université Paris-Est, 94700 Maisons-Alfort, France

**Keywords:** *Ornithodoros moubata*, histone methyltransferase, DOT1L

## Abstract

Epigenetic mechanisms have not been characterized in ticks despite their importance as vectors of human and animal diseases worldwide. Our investigation identifies and functionally characterizes the orthologue of S-adenosylmethionine (SAM) binding methyltransferase enzyme, disruptor of telomeric silencing 1-like (DOT1L) in *Ornithodoros moubata* (OmDOT1L), a soft tick vector for the relapsing fever pathogen *Borrelia duttonii* and the African swine fever virus. The OmDOT1L tertiary structure was predicted and compared to the *Homo sapiens* DOT1L which had been co-crystalized with SGC0946, a DOT1L-specific inhibitor. The amino acid residues crucial for SAM and SGC0946 binding conserved in most DOT1L sequences available, are also conserved in OmDOT1L. Quantitative PCR of *Omdot1l* during *O. moubata* life stages showed that transcripts were significantly upregulated in first-stage nymphs. *O. moubata* larvae exposed to SGC0946 displayed high mortality during molting to first-stage nymphs. Furthermore, a significant decrease in weight was observed in second-stage nymphs fed on recombinant OmDOT1L-immunized rabbits. In contrast, artificial blood feeding supplemented with SGC0946 did not affect survival and reproductive performance of adult female ticks. We concluded that OmDOT1L plays an essential role in the regulation of larval molting and the feeding of *O. moubata* second-stage nymphs.

## 1. Introduction 

Ticks are hematophagous ectoparasites and are the second most important vectors of human and animal pathogens following mosquitoes [1,2]. *Ornithodoros moubata*, a soft tick of the family Argasidae, is a vector of important pathogens such as *Borrelia duttoni* and African swine fever virus [1]. The lifecycle of *O. moubata* depends on multiple animal hosts and in contrast to hard ticks, it has two or more nymphal stages. Each nymphal stage requires a blood meal from a host [1]. *O. moubata* are rapid feeders with an average feeding time of one hour after which they drop off the host. The ability of *O. moubata* to feed rapidly is an important factor that influences the transmission of pathogens and spreading of diseases [1]. Regulation of the transcriptional programs involved in tick molting and feeding are poorly understood, but “*master switch*” mechanisms such as chromatin modulation and the epigenetic machinery might be involved.

Post-translational modifications (PTMs) of histones are an important component of epigenetic regulation of gene expression and they include methylation, phosphorylation, acetylation, ubiquitylation, and sumoylation [3]. PTMs mediate the epigenetic signaling that regulates most biological processes that are involved in the expression of DNA [3]. Enzymes responsible for histone PTMs are histone-modifying enzymes (HMEs) and these include histone deacetylases, methyltransferases, and lysine demethylases, among others. Histone methyltransferases (HMTs) catalyze the transfer of one, two, or three methyl groups to lysine and arginine residues of histone proteins and are separated into two classes. The first class is composed of enzymes with a conserved Su(var) 3–9, Enhancer-of-zeste and Trithorax (SET) domain. The second class consists of enzymes without the SET domain but with a conserved S-adenosylmethionine (SAM) binding pocket. There is a single HMT without the SET domain, the disruptor of telomeric silencing 1-like (DOT1L), and this protein has been identified in several organisms [4,5,6,7,8].

DOT1L methylates lysine residues in the globular core of histones and it is the only enzyme responsible for histone H3 lysine 79 (H3K79) methylation [9,10,11,12,13]. Knockout of *dot1l* in yeast, flies, and mice results in complete loss of H3K79 methylation [9,10,11,12]. DOT1L has been reported to play an important role in DNA damage and repair response in cancer cells [14], as well as in heterochromatin formation and embryonic development [11]. *Dot1l*-deficient mouse embryos developed abnormalities and succumbed between 9 to 10 days post coitum [11]. *Dot1l* knockdown in *Xenopus tropicalis* embryos had no effect on embryogenesis but lethality prior to metamorphosis was observed [15]. Wen et al. (2017) [16] studied *Xenopus* development and they were able to prove the role of DOT1L as an coactivator of thyroid hormone receptors. Furthermore, *Dot1l* is activated by thyroid hormone at the transcription level during *Xenopus* metamorphosis [17].

Tick epigenetics is still in its infancy. In a preliminary investigation, all major HMEs including histone acetyltransferases (e.g., IsHAT1, members of the MYST family and E1A-binding protein p300/CREB-binding protein), histone deacetylases (HDAC, e.g., Sirtuins, IsHDAC1, 3, 4 and 8), lysine specific histone demethylases (LSD, i.e., IsLSD1A and IsLSD1-B), and HMTs (e.g., SET domain containing HMTs and IsDOT1L) were identified in the genome of the hard tick *Ixodes scapularis* [18]. In this study, the DOT1L orthologue was identified and isolated in *O. moubata* (OmDOT1L) and functional studies were used to explore the role of OmDOT1L in tick molting, feeding, and reproduction.

## 2. Materials and Methods

### 2.1. Animals

*O. moubata* ticks were obtained from a laboratory colony maintained at the Institute of Parasitology, Biology Centre of the Czech Academy of Sciences (BC CAS), Ceske Budejovice, Czech Republic. Larvae, nymphs, and adult females were fed on New Zealand white rabbits and C3H/HeN mice. Except for on-host feeding, ticks were maintained in 50-mL plastic tubes with a 14:10 (light:dark) photoperiod, 25.0 ± 3 °C, and 80–85% relative humidity. All laboratory animals were treated in accordance with the Animal Protection Laws of the Czech Republic No. 246/1992 Sb., ethics approval issued by the ethical committees at the BC CAS, the State Veterinary Administration, and the Central Commission for Animal Welfare under protocol No. 138/2016.

### 2.2. Primers Design, Gene Amplification, Cloning, and Sequencing of Omdot1l

The ticks were washed with 70% ethanol and dried on filter paper before RNA extraction. RNeasy®Mini kit (QIAGEN GmbH, Hilden, Germany, Cat. No.74104) was used for total RNA extraction following the recommendations of the manufacturer. First strand cDNA was synthesized using 500 ng of total RNA, Oligo(dT) primers, and Invitrogen Superscript™ III Reverse transcriptase (Thermo Fisher Scientific, Waltham, MA, USA, Cat. No. 18080044) kit as per manufacturer’s recommendations. 

*Omdot1l* coding sequence specific primers were designed from conserved regions of the aligned nucleotide sequences of this gene in *I. scapularis* (*Isdot1l*, GenBank database ID: XP_002403964), *Ornithodoros rostratus* (*Ordot1l*, GenBank database ID: GCJJ01002031), and *Ornithodoros turicata* (*Otdot1l*, GenBank database ID: GGLE01001146). *O. rostratus* and *O. turicata dot1l* sequences were retrieved from NCBI Transcriptome Shotgun Assembly Sequence Database by performing tblastn [19] using the amino acid sequence of IsDOT1L as query. Multiple alignment software MAFFT version 7 [20] was used to align the three sequences with default settings. Primers were designed using Primer 3 online software [21,22] and the forward and reverse primers resulted as follows: Dot1L-F ‘TACAACACTGCAGTCACCGATC’ and Dot1L-R ‘AATAACATGGAGGTAGTAAGAGACTGG’.

PCR amplification was performed using a PCR reaction of 50 µL that contained 25 µL of 1X master mix containing reaction buffer, Taq polymerase and dNTPs (Thermo Fisher Scientific, Waltham, MA, USA, Cat. No. K0171), 2 µL of each primer, Dot1L-F and Dot1L-R, at a final concentration of 0.25 µM each, 2 µL of cDNA (20 ng), and 19 µL of Milli-Q water. The amplification program was set with a denaturation at 94 °C for 2 mins, followed by 35 cycles of 94 °C for 30s, annealing temperature (as for respective primers) for 30s, and extension at 72 °C for 1 min, with a final extension step of 3 mins.

The amplicon of expected size was electrophoresed in 1.2% agarose gel and extracted and purified using QIAquick PCR & Gel Cleanup kit (QIAGEN GmbH, Hilden, Germany, Cat. No. 28506). The purified PCR product was cloned in pCR™2.1-TOPO vector using Invitrogen TOPO TA Cloning kit (Thermo Fisher Scientific, Waltham, MA, USA, Cat. No. K450002) according to the manufacturer’s protocol. The plasmid was isolated using NucleoSpin Plasmid Mini Prep kit (Macherey-Nagel, Düren, Germany, Cat. No. 740588.250). Colony PCR was performed using vector primers M13F and M13R and ten PCR-positive plasmids were sent for sequencing at GATC Biotech, Konstanz, Germany.

### 2.3. Quantitative PCR

In order to quantify *Omdot1l* gene expression, the total RNA was extracted from eggs, unfed and fed larvae, unfed and fed first-stage nymphs, and unfed adult males and females. RNA was quantified using Nanodrop 2000 Spectrophotometer (Thermofisher Scientific, Waltham, MA, USA). An equal quantity of RNA (100 ng) was used to synthesize cDNA using Super Script III reverse transcriptase enzyme (Thermo Fisher Scientific, Waltham, MA, USA, Cat. No. 18080044). Quantitative PCR (qPCR) gene-specific primers were designed using Primer 3 online software [21,22]. The following primers were obtained: realDOTL1-F ‘ACGATGGACAGCAACTTTCG’ and realDOTL1-R ‘CCTGTGGACCTCACTGAGAA’. The *O. moubata* ribosomal protein S4 (*rps4*) gene was used as the endogenous control gene [23]. qPCR amplification efficiencies were determined before using the primer set for the qPCR assays. 

qPCR reactions were carried out in a Light Cycler 480 (Roche, Basel, Switzerland). FastStart Universal SYBR Green Master Mix (Rox) (Sigma-Aldrich, St. Louis, Missouri, USA Cat. No. 4913850001) and an equal amount of cDNA (10 ng) per sample were used in 20 μl reactions that contained 0.3 μM of each primer. A melting curve analysis to verify primer dimers and non-specific amplifications was performed. The CT values were recorded and 2^-ΔΔCt^ method [24] was used to calculate the relative expression values. Three technical replicates were analyzed. The statistical significance between groups was evaluated using the Unpaired *t* test in the GraphPad 6 Prism program (GraphPad Software Inc., San Diego, CA, USA). Differences were considered significant when *p* < 0.05.

### 2.4. Phylogenetic Tree

DOT1L amino acid sequences from several taxonomic groups namely Chelicerata (8 species), Fungi (6), Amphibia (3), Mammalia (10), Holocephali (1), Euteleostomi (2), Insecta (42), Collembola (1), and Actinopterygii (4), were collected from GenBank. The catalytic domain of DOT1L was trimmed from the rest of the sequence and it was then used for subsequent analysis. Sequences were aligned using MAFFT configured for highest accuracy [20] and non-aligned regions were removed with Gblocks (v 0.91b) [25]. The final alignment had 201 gap-free amino acid positions. The best-fit model of the sequence evolution was selected based on Akaike Information Criterion (AIC) and Bayesian Information Criterion (BIC) implemented in Molecular Evolutionary Genetics Analysis (MEGA) v.6.00 [26]. The LG model [27], which had the lowest values of AIC and BIC, was chosen for subsequent phylogenetic analyses. Maximum likelihood (ML) method, implemented in MEGA, was used to obtain the best tree topologies. A proportion of Gamma distributed sites (G) was estimated in MEGA. Reliability of internal branches was assessed using the bootstrapping method (1000 bootstrap replicates) implemented in MEGA. Graphical representation and editing of the phylogenetic tree was performed with MEGA.

### 2.5. Tertiary Structure Prediction 

The OmDOT1L protein sequence was submitted to the Robetta server [28] to predict the tertiary structure. The Robetta server uses a comparative-based approach by parsing putative domains and structural models in several protein databases [28]. All predicted structures were superpositioned with the *Homo sapiens* DOT1L [13] using the Schrodinger’s Maestro sequence-based structural alignment plug-in [29]. The top OmDOT1L structure was selected based on its similarities to the *H. sapiens* DOT1L [13] alpha-carbon backbone and global folding. The predicted binding sites with inhibitor SGC0946 were based on the interacting residues of the co-crystalized 5-iodotubercidin with *H. sapiens* DOT1L [13]. 

### 2.6. Recombinant OmDOT1L Expression

The OmDOT1L catalytic domain cloned in the pCR™2.1-TOPO vector was re-amplified with the primers D1LProt-F—GACTCGAGTACAACACTGCAG and D1LProt-R—CCAAGCTTAATAACATGGAGG that included *XhoI* and *Hind III* restriction sites (underlined in the primer sequences), respectively. The amplified fragment was double-digested with the restriction enzymes *XhoI* (NEB, Massachusetts, USA, Cat. No. R0146S) and *Hind III* (NEB, Massachusetts, USA, Cat No. R0104S), purified and cloned in a pBAD/His A vector containing a N-terminal polyhistidine (6xHis) tag (Thermofisher Scientific, Waltham, MA, USA, Cat. No. V43001). Previous to the ligation using T4 DNA Ligase (Thermofisher Scientific, Waltham, MA, USA, Cat. No. EL0011), the pBAD/His A vector was also double-digested with *XhoI* and *Hind III* restriction enzymes. *Escherichia coli* BL21 competent cells were transformed with the recombinant vector. 

The protein synthesis was induced with 0.2% L-(+) arabinose at 37 °C. The bacterial cells were harvested 4 h after induction. The cell pellet was homogenized by sonication in 25 Mm Tris (pH 8.0), 300 mM NaCl buffer, and centrifuged at 8000 rpm for 10 mins at 4 °C. As the recombinant (r) protein rOmDOT1L was not present in the soluble fraction of the sonicated cell pellet, we proceeded to protein extraction from the cell pellet. After centrifugation, the cell pellet was dissolved overnight in 25 Mm Tris (pH 8.0), 300 mM NaCl buffer containing either 0.2% of the detergent N-lauroyl sarcosine or 8 M urea. The dissolved cell pellet was centrifuged and the supernatant containing the soluble fraction was used for downstream purification of rOmDOT1L. The supernatant was loaded on Protino Ni-NTA agarose columns (Macherey Nagel, Duren, Germany, Cat no. 745400.25) with shaking for 1 h at 4 °C to facilitate binding of the His-tagged recombinant protein to the agarose. Two washing steps were performed with a buffer containing 25 Mm Tris (pH 8.0), 300 mM NaCl, 0.2% N-lauroyl sarcosine, and 20 mM imidazole. The protein was eluted from the Ni beads with 25 Mm Tris (pH 8.0), 300 mM NaCl buffer, 0.2% N-lauroyl sarcosine, and 500 mM Imidazole. Desalting and protein concentration was achieved using a Amicon Ultra-4 Centrifugal Filter Unit (Merck, MA, USA, Cat. No. UFC800308) following manufacturer’s recommendations.

### 2.7. Detection of rOmDOT1L by Western Blot 

Protein samples (10 µg) were mixed with a loading buffer (2X) containing Sodium dodecyl sulfate (SDS), heated to 100 °C and applied to a 12% polyacrylamide gel. Following the polyacrylamide gel electrophoresis (PAGE), protein profiles were visualized using Coomassie Brilliant Blue R-250 staining (Bio-Rad Laboratories, Hercules, CA, USA, Cat. No. 160435). Parallel to this set up, another SDS-PAGE gel was run, and the proteins were transferred to Immun-Blot LF PVDF membrane (Bio-Rad Laboratories, Hercules, CA, USA, Cat. No. 1620177) using Trans-Blot Turbo system (Bio-Rad Laboratories, Hercules, CA, USA). Before transferring the proteins, membranes were blocked with 2.5% (*w*/*v*) non-fat skimmed milk dissolved in Phosphate-buffered saline (PBS) with 0.05% Tween 20 (PBST). A His-Tag horseradish peroxidase-conjugated antibody (R&D systems, MN, USA, Cat. No. MAB050H) was diluted in PBST (1:1000) and used for binding and detection of His-tagged rOmDOT1L. The membranes were washed three times with 1X PBST for 15 mins. Pierce ECL Western Blotting kit (Thermo Fisher Scientific, Waltham, MA, USA, Cat. No. 32106) was used for chemiluminescent assay and the signals were analyzed in Chemi Doc Imager (Bio-Rad Laboratories, Hercules, CA, USA).

### 2.8. Rabbit Immunization and Tick Infestation

For rabbit immunization, the purified rOmDOT1L was resuspended in PBS and mixed with incomplete Freund’s adjuvant (1:1 ratio) to a final protein concentration of 100 μg/mL. A mock preparation included sterile PBS and incomplete Freund’s adjuvant (1:1 ratio). Two independent experiments including two rabbits each were performed. In each experiment, rabbits were injected s. c. with three doses (weeks 1, 3 and 6) of the mix rOmDOT1L/adjuvant or the mock preparation for control infestations. Two weeks after the last immunization, rabbit blood samples were taken from the ear vein and checked for the presence of anti-rOmDOT1L antibodies using rOmDOT1L in a Western blot. Three weeks after the last immunization, and following the confirmation of the presence of anti-rOmDOT1L antibodies in rOmDOT1L-immunized rabbits, animals were infested with 21-27 *O. moubata* second-stage nymphs per animal. Engorged nymphs were collected, counted, weighted, and kept separately in 50 mL plastic tubes in controlled conditions. The statistical significance between groups was evaluated using the Unpaired *t* test in the GraphPad 6 Prism program (GraphPad Software Inc., San Diego, CA, USA). Differences were considered significant when *p* < 0.05. 

### 2.9. Membrane Feeding of Adult Females O. moubata 

Membrane feeding of the ticks was performed as described by Krull et al. 2017 [30] with modifications as in Knorr et al. 2018 [31]. Female *O. moubata* were fed on bovine blood with and without the DOT1L inhibitor SGC0946 (Sigma-Aldrich, St. Louis, MO, USA, Cat. No. SML1107), which is a highly DOT1L-specific inhibitor (*K_D_* of 0.06 nM and IC_50_ 0.3 ± 0.1 nM) that binds to the SAM binding site blocking the enzymatic activity of DOT1L [13]. Blood was supplemented with gentamycin (5μg/mL) and 1mM adenosine 5′-triphosphate (ATP). SGC0946 was dissolved in 50% dimethyl sulfoxide (DMSO) and added in the blood to a final concentration of 0.02 μM. Control ticks were fed with blood containing the same volume of 50% DMSO but without SGC0946. The ticks were placed in the feeding units which contained 3.1 mL of bovine blood previously warmed at 37 °C. After engorgement, the ticks were kept in 50 mL plastic tubes at 25.0 ± 3 °C, and relative humidity of 80–85% throughout the preoviposition and oviposition periods. Eggs laid by females were kept in the same conditions until larval hatch. Then, the mortality, percentage of egg laying, time to oviposition, amount of eggs per female, and percentage of egg hatching per egg batch were monitored, quantified, and compared between SGC0946-treated and control group. This experiment included three biological replicates with five ticks per group in each replicate. The statistical significance between groups was evaluated using the Unpaired *t* test and Chi-squared (χ^2^) test in the GraphPad 6 Prism program (GraphPad Software Inc., San Diego, CA, USA). Differences were considered significant when *p* < 0.05. 

### 2.10. Larval Immersion Test Using the DOT1L Inhibitor SGC0946

The inhibitor SGC0946 was used in a larval immersion test (LIT) using a protocol established for testing acaricides [32,33,34]. Four groups of 60 *O. moubata* unfed larvae each were included in this assay, one control group treated with DMSO and three experimental groups treated with different concentrations of SGC0946, 0.1 μM, 1 μM, and 10 μM. Larvae were immersed for 15 mins in the control and inhibitor solutions, then dried using a filter paper and incubated at 25.0 ± 3 °C, and 80–85% relative humidity [32,33,34]. After the LIT, half of the larvae of each group were fed on mice and fully engorged larvae were collected and left to molt to first-stage nymphs for three weeks in controlled conditions of temperature and humidity. The other half of the larvae was left unfed. Three weeks after the ticks had dropped from the mice, at least 90% of the larvae in the control group had molted to first-stage nymphs and the experiment was stopped. Larval pupa that had not finished molting within the three-week period were considered as dead. Percentage mortality was calculated for each of the above groups and the statistical significance of the difference in percentage mortality between SGC0946-treated and control groups was evaluated using the Chi-squared (χ^2^) test in the GraphPad 6 Prism program (GraphPad Software Inc., San Diego, CA, USA). Differences were considered significant when *p* < 0.05. 

## 3. Results and Discussion

### 3.1. Phylogenetic and Molecular Characterization of OmDOT1L Catalytic Domain

**Figure 1:**
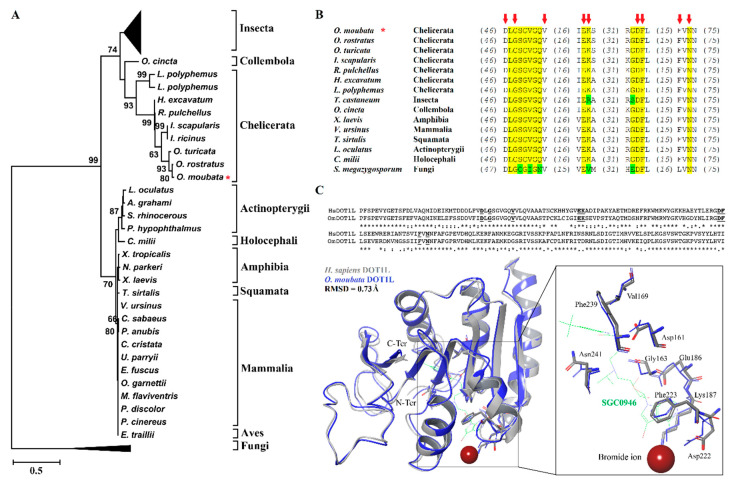
Phylogenetic and structural characterization of OmDOT1L. The evolutionary history of DOT1L was inferred by using the maximum likelihood method based on the LG matrix-based model. The tree with the highest log likelihood (−5402.58) is shown. Initial trees for the heuristic search were obtained automatically by applying neighbor-joining and BioNJ algorithms to a matrix of pairwise distances estimated using a LG model, and then selecting the topology with superior log likelihood value. The tree is drawn to scale, with branch lengths measured in the number of substitutions per site. The analysis involved 77 amino acid sequences and there were a total of 201 gap-free positions in the final dataset. Numerals close to the branches are bootstrap values. For display simplification, clusters including Insecta and Fungi DOTL1 sequences were collapsed. The position of OmDOT1L is shown with a red asterisk (*). A full version of the tree is available as Supplementary Figure S1 (**A**). Multiple sequence alignment showing conserved (labeled in yellow) and less conserved (labeled in green) residues of S-adenosylmethionine (SAM) binding sites across DOT1L orthologues and the amino acid positions identified to interact with SGC0946 (red arrows). The sequence of OmDOT1L is shown with a red asterisk (*) (**B**). The alignment shows the catalytic domain of DOT1L from *H. sapiens* (Hs) and *O. moubata* (Om) with SGC0946 interacting residues underlined in bold. The superposition of the two DOT1L structures (Hs, grey and Om, blue) with the bromide ion (amber sphere), SGC0946 (green) and interacting residues shown. The amino-terminus (N-Ter) and carboxyl-terminus (C-Ter) are labeled. The inset shows a closeup of the active site with the bromide ion, SGC0946 and the interacting residues labeled (**C**).

The cDNA transcript of the cloned catalytic domain of *Omdot1l* (609bp, GenBank database ID: MF431592) shares 85.55% and 86.21% sequence identity with its orthologous sequences in the soft ticks *O. turicata* and *O. rostratus*, respectively. The translated amino acid sequence of OmDOT1L showed high identity with *O. turicata* (96.06%), *O. rostratus* (98.03%), and *I. scapularis* (87.19%) sequences. The theoretical molecular weight of the catalytic domain was estimated to be 23 kDa. The amino acid sequences of OmDOT1L orthologues from different taxa were aligned and used to study their phylogenetic relationship. The ML phylogenetic tree (Figure 1A) reveals a close phylogenetic relationship between the major taxonomic groups of DOT1L protein sequences. An extended version of this tree is available as Supplementary Figure S1. Interestingly, available Ixodidae DOT1L sequences of *I. scapularis* and *Ixodes ricinus* did not cluster with the other ticks of the same family, i.e., *Hyalomma excavatum* and *Rhipicephalus pulchellus*. Instead, *I. scapularis* and *I. ricinus* DOT1L sequences clustered with Argasidae ticks DOT1L sequences. All available *Ornithodoros* DOT1L sequences clustered together.

Conserved domain search resulted in the identification of the 13 conserved residues of SAM binding sites in the catalytic domain of OmDOT1L (Figure 1B). These residues were also highly conserved in the 77 taxa included in the phylogenetic tree (Supplementary Figure S2). This suggests that these residues are critical for SAM binding and DOT1L enzymatic activity.

The primary sequence and predicted tertiary structure of OmDOT1L was further investigated for specific binding mechanisms to SAM analogues. OmDOT1L is 70.7% identical to the *H. sapiens* DOT1L (HsDOT1L) (Figure 1C). The structure of HsDOT1L co-crystalized with the SAM brominated analogue inhibitor, SGC0946, is available [13]. The specific HsDOT1L-SGC0946 binding residues are conserved in OmDOT1L suggesting a similar inhibitory mechanism (Figure 1C). Superposition of the HsDOT1L and OmDOT1L structures shows the high conservation of secondary and tertiary folds with an α-carbon backbone root mean square deviation (RMSD) of 0.73 Å. Because of this high conservation, the coordinating residues of SGC0946 and the bromide ion are similarly oriented in both DOT1Ls (Figure 1C), more specifically, the pi–pi stacking of Phe223 and the adenine ring of SGC0946. The Phe223 belongs to the hydrophobic cleft of DOT1L that determines the potency of inhibitors [13]. Results showed that key functional amino acid residues and the tertiary structure of the H3K79 histone methyltransferase DOT1L are conserved in the *O. moubata* orthologue OmDOT1L. The SGC0946 binding residues and the conformation of their side chains were also conserved in OmDOT1L. This high structural conservation supports OmDOT1L being a functional H3K79 histone methyltransferase and that SGC0946 is a potential inhibitor of this enzyme in *O. moubata*. This notion, however, remains to be confirmed experimentally with enzymatic assays in which SGC0946 can be used to inhibit the methyltransferase activity of rOmDOT1L in vitro.

### 3.2. Omdot1l Gene is Upregulated in Unfed First Stage Nymphs

**Figure 2:**
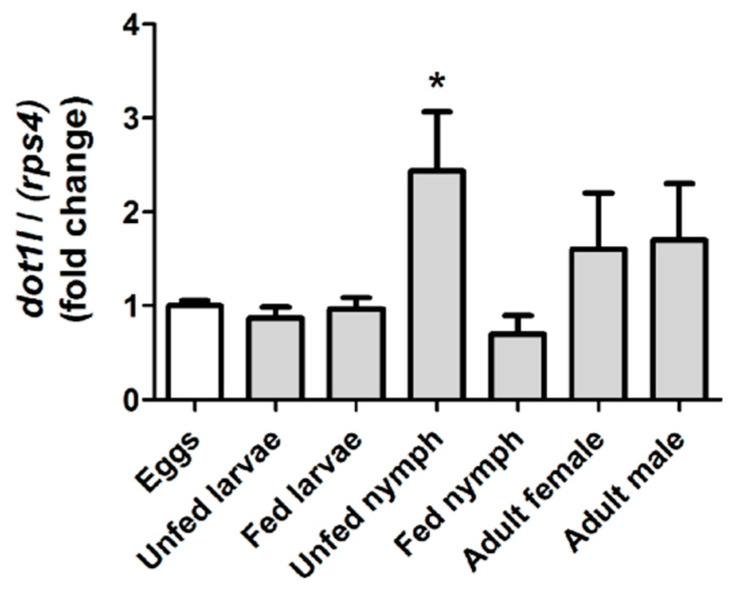
Relative expression of *Omdot1l* in different lifecycle stages of *O. moubata*. The relative expression of *Omdot1l* was determined using qPCR. Total RNA was extracted from eggs (white bar), unfed and fed larvae, unfed and fed first-stage nymphs and adult male and female *O. moubata* (grey bars). cDNA was synthesized and equal amounts of cDNA were used in a qPCR assay using *rps4* as a normalizing gene. The expression of mRNA is relative to the expression of *Omdot1l* in the eggs. Unpaired *t* Test: * *p* < 0.05.

Gene expression analysis of *Omdot1l* was performed for various stages of *O. moubata* lifecycle including eggs; fed and unfed larvae; fed and unfed first-stage nymphs and adult male and female ticks. Relative expression values of *Omdot1l* gene expression in all stages were calculated relative to the expression of the gene in eggs (Figure 2). Significant upregulation of *Omdot1l* gene expression was observed in unfed first-stage nymphs. *Omdot1l* gene expression in unfed first-stage nymphs was more than 2-folds higher than that in eggs, unfed and fed larvae and fed first-stage nymphs. Although with a tendency to increase, there was no significant difference in gene expression in male and female ticks compared to *Omdot1l* expression in eggs. The gene expression analysis suggests that OmDOT1L may have an important role in unfed first-stage nymph compared to the other stages of *O. moubata* lifecycle. 

### 3.3. The DOT1L Inhibitor SGC0946 Reduces Larval Molting Success in O. moubata

**Figure 3:**
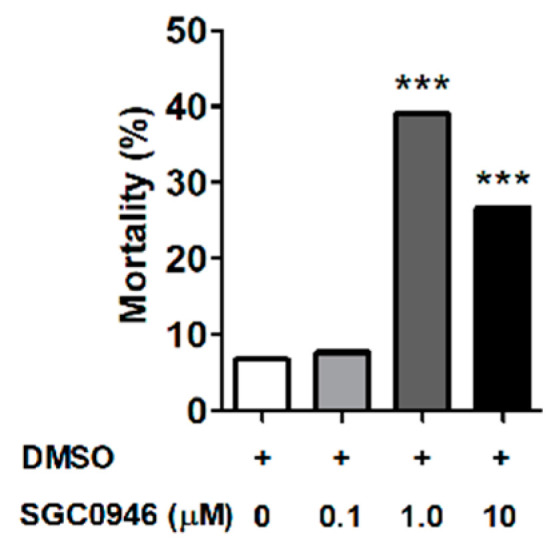
Effect of inhibitor SGC0946 on *O. moubata* larval molting. Larvae were immersed for 15 min either in DMSO (+, control), or the inhibitor SGC0946 (dissolved in DMSO) at three different concentrations, 0.1 μM, 1 μM, and 10 μM. Larvae were then dried on a filter paper, fed on mice and fully fed larvae were left to molt to first-stage nymphs in controlled conditions. The percentage of larval mortality during molting was calculated for each SGC0946-treated group and compared with the control. Chi-squared (χ^2^) test: *** *p* ≤ 0.002.

As DOT1L was previously reported to regulate *Xenopus* metamorphosis [17], we hypothesized that *Omdot1l* upregulation in unfed first-stage nymphs compared with fed larvae was due to a functional role of OmDOT1L in *O. moubata* molting. To test this hypothesis, we used the DOT1L-specific inhibitor SGC0946 in a larval immersion test (LIT) assay using *O. moubata* larvae. Four groups of 60 larvae each were immersed in different concentrations of SGC0946 (i.e., 0.1 μM, 1 μM, and 10 μM) and DMSO as control. After 15 mins of immersion, half of the larvae (*n* = 120, 30 per group) were placed at 25.0 ± 3 °C, and 80–85% relative humidity to evaluate unfed larval mortality, and the other half (*n* = 120, 30 per group) was allowed to feed on mice to evaluate mortality associated with feeding and/or molting. Fully engorged larvae were collected and incubated in controlled conditions of temperature and humidity for molting. Mortality of unfed larvae, fed larvae, and molting larvae at each SGC0946 concentration was recorded and compared with the control groups. No significant mortality was observed in unfed or feeding larvae treated with any of the tested SGC0946 concentrations (data not shown). In addition, the percentage of larval molting mortality at 0.1 μM SGC0946 was comparable to that of the control group (Figure 3). 

However, a significant increase in the percentage of larval molting mortality was observed when 1 μM (χ^2^ = 30.29, df: 3, *p* = 0.0001) and 10 μM (χ^2^ = 14.17, df: 3, *p* = 0.002) SGC0946 were used in the LIT assay (Figure 3). The larval molting mortality at 1 μM and 10 μM suggests that SGC0946 inhibits OmDOT1L which may be involved in the regulation of tick molting. This result is in accordance with a previous report in *Xenopus* where DOT1L was shown to be critical for *Xenopus* development and metamorphosis [16,17]. Another complementary investigation showed that *dot1l* knockout in *Xenopus* embryos did not result in failure of hatching or embryogenesis, but in tadpole mortality presumably due to retarded growth prior to metamorphosis [15]. The methylation of H3K79 (H3K79me), exclusively catalyzed by DOT1L [6,7], is an active chromatin marker and is prominent in actively transcribed regions of the genome [4,5,6,7,8]. Since high concentrations of SGC0946 induce larval mortality during molting, we postulate that OmDOT1L might facilitate larval molting. SGC0946 is highly selective for DOT1L and was inactive against a panel of 12 HMTs and DNMT1 [13]. SGC0946 potently reduces H3K79me in cells with IC_50_ < 9 nM [13]. Inhibition by SGC0946 might reduce the enzymatic activity of OmDOT1L which in turn might reduce the levels of H3K79me in the globular core of tick histones. Reduction of H3K79me can potentially impact the regulation of telomeric silencing, cellular development, cell-cycle checkpoint, DNA repair, and regulation of transcription [11,14,15,16].

### 3.4. Immunity against Recombinant OmDOT1L in Rabbits Impairs Second-Stage Nymphal Feeding

On the basis of the high levels of *Omdot1l* expression in unfed first-stage nymphs (Figure 2), and the larval molting mortality associated with SGC0946 (Figure 3), we asked whether OmDOT1L was involved in the performance of *O. moubata* second-stage nymph. To test this hypothesis, the mortality during feeding, weight after feeding, mortality during molting, and weight after molting were monitored in second-stage nymphs fed on rabbits immunized with recombinant OmDOT1L (rOmDOT1L). 

**Figure 4:**
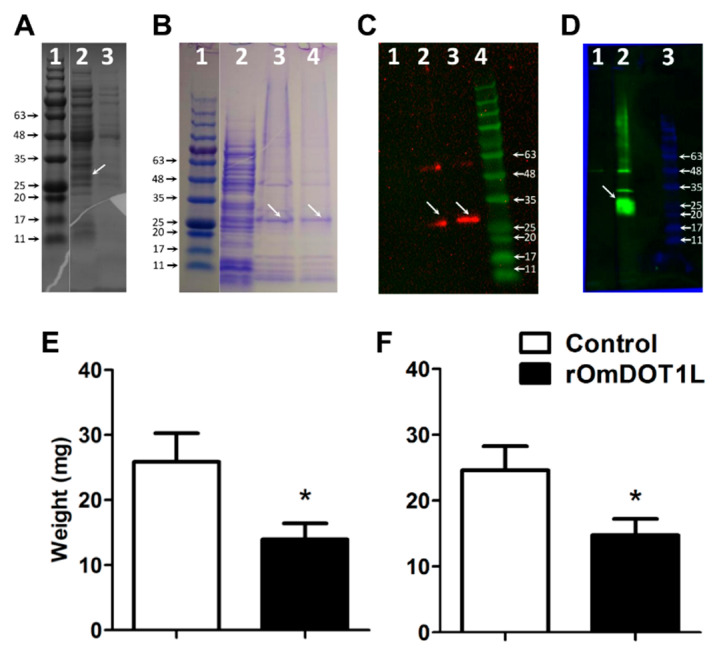
Effect of rOmDOT1L immunization on *O. moubata* feeding on rabbits. The rOmDOT1L protein of the expected size (approx. 27 kDa) was identified in the cell pellet of *Omdot1l*-transformed *E. coli*; lane 1 molecular weight marker, lane 2 protein extract of *Omdot1l*-transformed *E. coli* (arrow shows rOmDOT1L), and lane 3 protein extract of control non-transformed *E. coli* (**A**). Ni-NTA purification of n-lauryl sarcosine or 8 M urea solubilized *E. coli* pellet; lane 1 molecular weight marker, lane 2 crude protein extract of *Omdot1l*-transformed *E. coli,* and lanes 3 and 4 purification products using n-lauryl sarcosine and 8 M urea, respectively (arrows show rOmDOT1L) (**B**). Anti-His tag antibodies revealed the presence of a single band in the protein extract purified with 8 M urea; lane 1 negative control (i.e., non-transformed *E. coli*), lane 2 rOmDOT1L purified with n-lauryl sarcosine, lane 3 rOmDOT1L purified with 8 M urea, and lane 4 molecular weight marker (**C**). The presence of antibodies raised against rOmDOT1L protein in rabbit were confirmed by Western blot (**D**). The average weight of the second-stage nymphs post-feeding (**E**) and post-molting (**F**) decreased significantly compared with the control group. Student’s t-test: * *p* < 0.05). Results are representative of two biological replicates.

The recombinant protein was obtained in *E. coli* using the pBAD/His A-based expression system. A protein of the expected size (approx. 23kDa of protein fragment + 3 kDa tag) was identified in the cell pellet using either 0.2% N-lauroyl sarcosine or 8 M urea for solubilization (Figure 4A). 

Solubilized protein extracts were successfully purified using Ni-NTA column (Figure 4B). A protein fraction with less contaminants resulted from the protein solubilized in 8 M urea (Figure 4B). Anti-His tag antibodies were used to identify His-tagged rOmDOT1L in a Western blot. Only one band of the expected size was observed in the Western blot of the protein pellet solubilized with 8 M urea, whereas two bands were observed in the protein pellet solubilized with 0.2% N-lauroyl sarcosine (Figure 4C). Therefore, only the protein solubilized in 8 M urea was used for downstream immunizations. The purified rOmDOT1L was used to immunize rabbits and, two weeks after the last immunization, the production of anti-rOmDOT1L antibodies was confirmed by Western blot (Figure 4D). 

Three weeks after the last immunization, *O. moubata* second-stage nymphs (*n* = 21–27 per group) were fed either on the rOmDOT1L-immunized or the control rabbits. Fully engorged second-stage nymphs were counted, weighted and kept in controlled conditions to quantify the percentage of nymphs that molt to the next nymphal stage as well as their weight after molting. A clear reduction in the weight of second-stage nymphs after feeding (Figure 4E) and after molting (Figure 4F) was observed in the rOmDOT1L-immunized group. No mortality of second-stage nymphs was associated with rOmDOT1L immunization.

Host immunization has been used previously for the functional characterization of tick proteins localized in the cytoplasm and nucleus of tick cells [35,36]. The rationality of this approach in ticks is based in the capacity of tick cells to uptake fully functional host immunoglobulins [37]. Once internalized by tick cells, host immunoglobulins retain their full antigen-binding activity reaching intracellular targets. DOT1L is a nuclear protein and the results suggest that rOmDOT1L immunization induces the generation of rOmDOT1L-specific antibodies that may impair the cellular function of native OmDOT1L during the feeding of second-stage nymphs. Tick feeding and blood digestion are associated with changes in the expression of thousands of *O. moubata* midgut genes [38]. Whether midgut gene expression regulation during feeding involves epigenetic mechanisms remains to be tested. However, the presence of most HMEs in ticks [18], and the impairment of *O. moubata* feeding in ticks that fed on rOmDOT1L-immunized rabbits suggest that H3K79me may account as one of the epigenetic mechanisms involved in gene expression activation during feeding in second-stage nymph.

### 3.5. The DOT1L Inhibitor SGC0946 Does not Affect the Reproductive Performance of Adult Females O. moubata 

**Figure 5:**
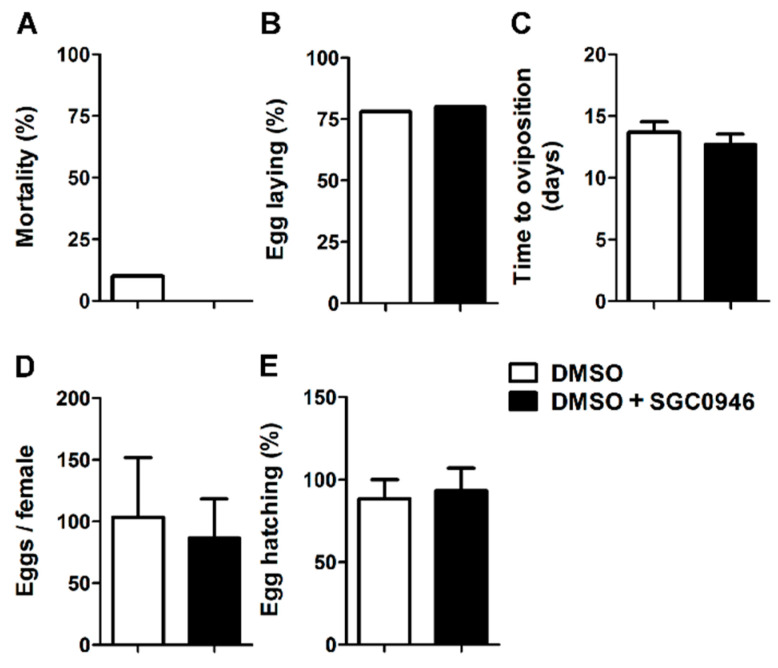
Effect of inhibitor SGC0946 in adult female *O. moubata*. Ticks were placed in an artificial feeding system and allowed to fed until repletion with bovine blood. The blood, with and without the inhibitor SGC0946 dissolved in DMSO was supplemented with gentamycin and ATP as feeding stimulant. The final concentration of the inhibitor in the blood was 0.02 μM. After engorgement, the ticks were kept in controlled conditions of humidity and temperature and adult female mortality (**A**) egg laying (**B**), time to oviposition (**C**), eggs per female (**D**), and egg hatching (**E**) were monitored. Chi-squared (χ^2^) test was used for comparison in (A), (B), and (E) and unpaired *t* test was used for comparison in (C) and (D). No significant difference between the inhibitor and the control groups was observed for any of the parameters, *p* > 0.05.

We then asked whether in addition to playing a role in the molting and feeding of early stages of *O. moubata*, OmDOT1L was also involved in the survival and reproductive performance of adult female *O. moubata*. To answer this question, adult females *O. moubata* were fed in an artificial feeding system using bovine blood with and without SGC0946. *O. moubata* females were fed artificially to repletion with blood containing 0.02 µM SGC0946, and several parameters including mortality, oviposition, and egg hatching were monitored in the SGC0946-treated and DMSO control groups. No significant differences were observed in the percentage of adult tick mortality (Figure 5A), percentage of *O. moubata* females that laid eggs (Figure 5B), time to oviposition (Figure 5C), amount of eggs laid per *O. moubata* female (Figure 5D) and the percentage of egg hatching per egg batch (Figure 5E).

The lack of effect of SGC0946 on the amount of eggs per female, the time to oviposition, and the percentage of egg hatching suggest that OmDOT1L plays no role in egg and embryo development in *O. moubata*. Chemical compounds that impaired oviposition and embryonic development in *Dermacentor reticulatis* induced death of all eggs early after oviposition or decreased percentage of larval hatch eggs [39]. The lack of effect of SGC0946 on egg development and larval hatch in *O. moubata* concurs with previous reports where the knockdown of *dot1l* has no observed effect on embryogenesis of *X. tropicalis* as injected eggs develop normally into feeding tadpoles [15]. Interestingly, the *dot1l*-knockdown tadpoles experienced growth retardation and died before the metamorphosis [15]. Further experiments should test whether the offspring of female *O. moubata* fed on blood with SGC0946 is able to molt to first-stage nymphs.

## 4. Conclusions

This is the first study on the functional characterization of a HMT in ticks. We identified an orthologue of DOT1L in the soft tick *O. moubata*, namely OmDOT1L. Conservation of key amino acid residues and the tertiary structure of the catalytic domain of OmDOT1L compared to the human orthologue, HsDOT1L, suggests that OmDOT1L is responsible for H3K79 methylation in *O. moubata*. It is generally assumed that proteins with conserved sequence and structure have the same function in different organisms. However, the function of proteins described in model organisms like mammals and insects may be different in evolutionarily distant species such as ticks. Three functional studies were performed in *O. moubata* with OmDOT1L: LIT assay with the DOT1L-specific inhibitor SGC0946, feeding of second-stage nymphs on rOmDOT1L-immunized hosts, and artificial membrane adult female feeding on blood containing a DOT1L-specific inhibitor SGC0946. We conclude that OmDOT1L is involved in larval development to first-stage nymphs and in the feeding of second-stage nymphs. However, our results do not support a role of OmDOT1L in egg development, larval egg hatching, and oviposition. Further research is needed to understand the role of OmDOT1L in the regulation of molting and feeding in *O. moubata*. Our results also open the possibility to use OmDOT1L as a basis to develop novel anti-tick interventions that could reduce tick populations and lead to a possible reduction in the tick-borne pathogenic diseases that they cause. 

## Supplementary Materials

The following are available online at https://www.mdpi.com/2076-393X/8/2/157/s1, Figure S1: Phylogenetic tree of DOT1L protein sequences of major taxonomic groups. OmDOT1L orthologues from different taxa were collected from GenBank and aligned. The best-fit model of the sequence evolution was predicted to be the LG-model. Maximum likelihood method was used to obtain the best tree topologies and a proportion of Gamma distribution sites was estimated. The reliability of internal branches was assessed using the bootstrapping method (1000 bootstrap replicates). Interestingly, not all Ixodidae DOT1L sequences clustered together, but some clustered with ticks of the Argasidae family, Figure S2: Alignment of the catalytic domain of DOT1L sequences included in the phylogenetic tree. Conserved, and less conserved, residues of S-adenosylmethionine (SAM) binding sites are highlighted in yellow and green, respectively.
