# Supplementary figures and images for: Histone Methyltransferase DOT1L Is Involved in Larval Molting and Second Stage Nymphal Feeding in Ornithodoros moubata
Source: Vaccines (Basel). 2020 Apr 1;8(2):157. doi: 10.3390/vaccines8020157 (PMC7349889; doi:10.3390/vaccines8020157)

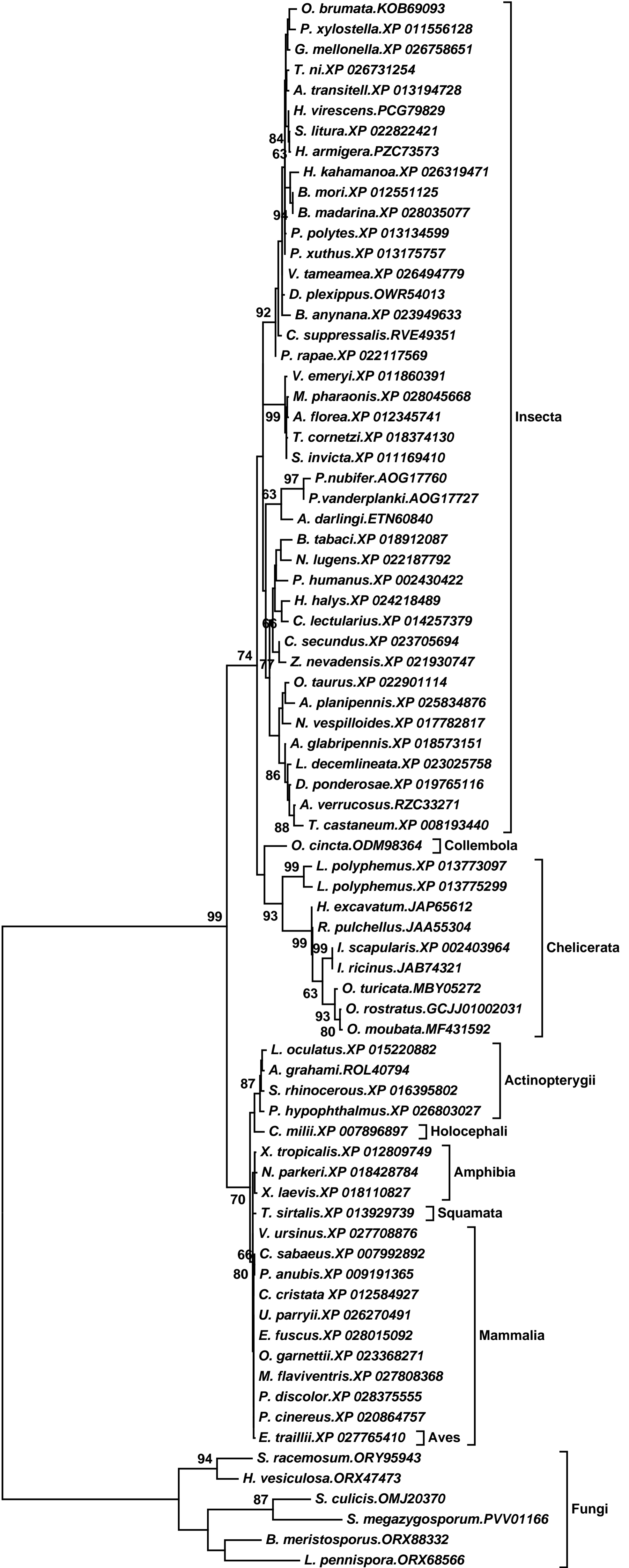

Supplement: Supplementary file 1 [file vaccines-08-00157-s001.zip › Supplementary Figure S1.pdf]

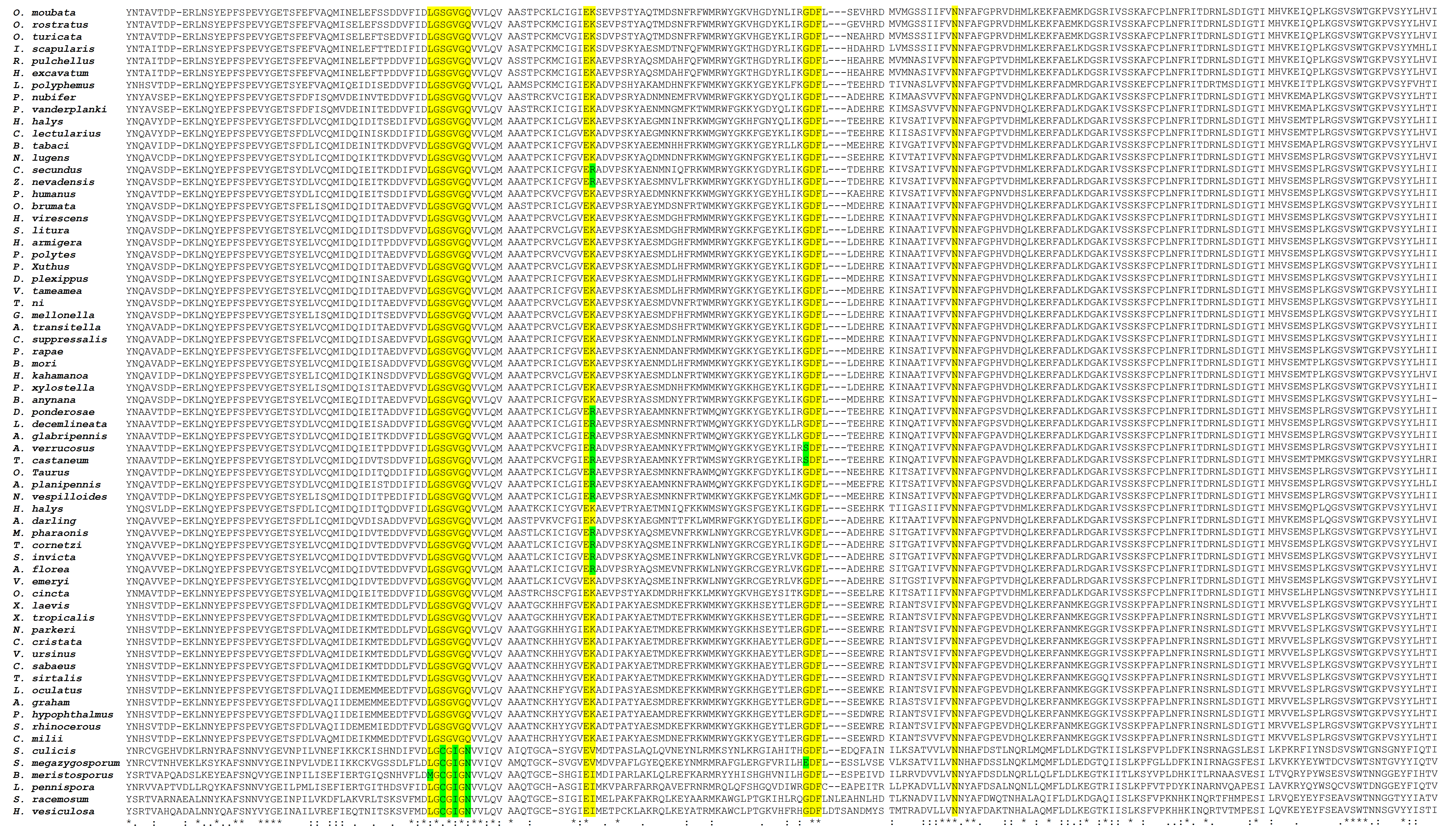

Supplement: Supplementary file 1 [file vaccines-08-00157-s001.zip › Supplementary Figure S2.tiff]
